# COVID-19 Detection and Diagnosis Model on CT Scans Based on AI Techniques

**DOI:** 10.3390/bioengineering11010079

**Published:** 2024-01-14

**Authors:** Maria-Alexandra Zolya, Cosmin Baltag, Dragoș-Vasile Bratu, Simona Coman, Sorin-Aurel Moraru

**Affiliations:** Department of Automatics and Information Technology, Transilvania University of Brasov, 500036 Brașov, Romania; cosmin.baltag@student.unitbv.ro (C.B.); dragos.bratu@unitbv.ro (D.-V.B.); simona.coman@unitbv.ro (S.C.); smoraru@unitbv.ro (S.-A.M.)

**Keywords:** CT scans, COVID-19, convolutional neural network, MobileNetV1

## Abstract

The end of 2019 could be mounted in a rudimentary framing of a new medical problem, which globally introduces into the discussion a fulminant outbreak of coronavirus, consequently spreading COVID-19 that conducted long-lived and persistent repercussions. Hence, the theme proposed to be solved arises from the field of medical imaging, where a pulmonary CT-based standardized reporting system could be addressed as a solution. The core of it focuses on certain impediments such as the overworking of doctors, aiming essentially to solve a classification problem using deep learning techniques, namely, if a patient suffers from COVID-19, viral pneumonia, or is healthy from a pulmonary point of view. The methodology’s approach was a meticulous one, denoting an empirical character in which the initial stage, given using data processing, performs an extraction of the lung cavity from the CT scans, which is a less explored approach, followed by data augmentation. The next step is comprehended by developing a CNN in two scenarios, one in which there is a binary classification (COVID and non-COVID patients), and the other one is represented by a three-class classification. Moreover, viral pneumonia is addressed. To obtain an efficient version, architectural changes were gradually made, involving four databases during this process. Furthermore, given the availability of pre-trained models, the transfer learning technique was employed by incorporating the linear classifier from our own convolutional network into an existing model, with the result being much more promising. The experimentation encompassed several models including MobileNetV1, ResNet50, DenseNet201, VGG16, and VGG19. Through a more in-depth analysis, using the CAM technique, MobilneNetV1 differentiated itself via the detection accuracy of possible pulmonary anomalies. Interestingly, this model stood out as not being among the most used in the literature. As a result, the following values of evaluation metrics were reached: loss (0.0751), accuracy (0.9744), precision (0.9758), recall (0.9742), AUC (0.9902), and F1 score (0.9750), from 1161 samples allocated for each of the three individual classes.

## 1. Introduction

The untimely inception of the theme under discussion derives from the existence of a class of viruses that follows a diversity principle and, as a result, it could be found both in humans and various animal species, termed coronavirus. Particularly, the SARS (Severe Acute Respiratory Syndrome) class emanated a pandemic that led to a rudimentary increased number of infected people, where about approximately 8000 Chinese patients [1] were subjected to the disease during the same period. Since 2004, no more such cases have been reported, until 2012, when a new coronavirus made its presence felt, developing the MERS (Middle East Respiratory Syndrome) class in this way. By 2019, approximately 2500 such cases have been announced with a fatality rate of 30% [2]. At the end of 2019, China reported a continued increase in such pneumonia cases in the city of Wuhan, Hubei Province, with such a nature that it could be determined as a new betacoronavirus in 2020, named SARS-CoV-2 by the International Committee for Taxonomy of Viruses, due to genetic analysis, rapidly spreading COVID-19 disease [3]. This novel disease, namely COVID-19, is cataloged as a major factor of an infectious pandemic by the World Health Organization (WHO). Concretely, the total global cases of over 30.6 million, claiming 950,000 lives, have made COVID-19 as prevailing, whereas nations have been looking for ways to contain its spread [4]. All these reports define the seriousness of the situation from the very beginning and derive the need for intervention in this context.

Frequently, lung infections caused by various viruses reveal common symptoms in the early stages of the disease, which can vary from one patient to another. The most common clinical manifestations of the infectious diseases of COVID-19 and influenza pneumonia are fever, dry cough, and fatigue [5,6], which denotes gravity in the possible states of the patients. Ideally, the mitigation of this severity comes from an early identification. At the current stage, different methods work on this mechanism, as confirmed by diagnosis. Although various PCR methods, such as RT, are the most specific methods for the diagnosis of viral pneumonia (their sensitivity is not optimal for all viruses). Nevertheless, the most recent studies are showing us continuity in the need for other additional methods, besides those previously specified [7].

**Figure 1:**
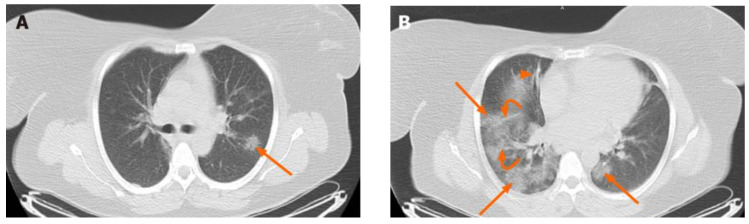
Chest radiographsof a 46-year-old female patient with fever and dry cough [9]. Axial chest computed tomography shows bilateral multifocal ground glass opacities (arrows; (**A**,**B**)), peribronchial interstitial thickening (arrowhead; (**B**)) and reticular opacities (curved arrows; (**B**)), consistent with coronavirus disease 2019 pneumonia.

From a strictly medical perspective, the idea of the seriousness of the patient’s condition is emphasized once the patient comes into contact with the current illness. More specifically, the main CT features are bilateral multifocal ground-glass pulmonary opacities overlapping consolidation, bronchiolar wall thickening, rounded opacities, and interlobular septal thickening [8]. In a specific case (Figure 1), the axial-computed tomography of the chest shows bilateral multifocal ground-glass opacities represented by red arrows, peribronchial interstitial thickening (arrow B), and reticular opacities (curved arrows; B). Pulmonary opacities with ground-glass appearance are most common and are mainly seen as bilateral and multifocal opacities with peripheral and posterior distribution. Pure consolidations are less common and may be seen later in the course of the disease or at older ages.

The stage immediately following the one of identifying the problem is constituted by a proposal of the solution. In this particular case, the root of the solution is the medical analysis. Recent studies have shown that chest CT has a high sensitivity for early detection of pneumonia [10,11,12,13]. Combining RT-PCR and chest CT may improve the sensitivity and specificity for diagnosing clinically suspected cases, such as COVID-19 [14,15]. This assortment, alongside the other diagnostic methods [16] for COVID-19 detection, is a common and effective approach. RT-PCR is widely used for detecting the presence of viral RNA, including that of the SARS-CoV-2 virus responsible for COVID-19. In other words, integrating multiple diagnostic techniques can enhance the overall accuracy, sensitivity, and reliability of COVID-19 testing.

Furthermore, the starting point, which, more precisely, ensures the pronouncement of a solution, is represented by the field of medical imaging [17]. This possesses an essential role in the diagnosis processes of patients, helping doctors to make decisions in this context. On the side of parallelism, as machines become more and more advanced and healthcare careers are in ongoing development, more and more data in the form of medical images are generated, among which computer tomographies are distinguished. Analyzing these huge amounts of medical data requires quite a long time for medical professionals. From a deeper perspective, this analysis mechanism can produce erroneous or biased results due to different degrees of experience, knowledge, and other factors that the experts themselves possess. In other words, the solution to the proposed problem can be extended to a higher level where state-of-the-art techniques are present, making a mapping from the medical area to the AI zone [18].

Therefore, a strategic approach is constituted by corroborating the problem raised with other domains. Deep learning (DL) techniques are often integrated into this field with promising results in various medical applications such as segmentation and registration of information from patient data [19]. DL is broadly used in various domains, such as finding abnormalities, object detection, and cataloging in the biomedical area using pre-trained or personalized models [20]. Considerable interest in the medical field has been given, especially to CNNs, proposing to solve problems associated with the segmentation part of medical imaging [21].

This paper proposes a model that can assist medical professionals in the rapid and accurate identification of consequences of lung diseases, namely COVID-19 and viral pneumonia, commencing from a perspective based on [22]. Simultaneously, it is desired to present a different approach to this topic via the meticulous technique of research, implementation, and analysis of the results. More precisely, this work includes several general current approaches, achieving a correlation between them in such a way as to arrive at valid and relevant results and to reflect a rich methodology. Moreover, besides the goal of proposing a solution to the addressed problem scrupulously, a didactic illustration at the approach level will be shown.

The emphasis is on a beginning that derives from the origin of the clinical data, which represents the foundation of the proposed solution. In the medical setting, computed tomography (CT) has proven to have a degree of effectiveness quite high compared to other clinical data of the patients, taking an accurate diagnosis as the point of view [23]. Accordingly, there is a desire to develop and conceive diverse methods to combat these negative effects. Among the many existing methods, artificial intelligence applications based on deep learning stand out. Thus, integrating DL techniques in the medical imaging field can solve many shortcomings.

From an architectural point of view, regarding the current piece of work, the structure of the paper is divided into four important points. The chapter associated with the introduction involves, in the first instance, a short history that shows how the beginning of the topic under consideration appeared, wanting to emphasize the need for continuous involvement in this subject. This new emerging problem is correlated with medical benchmarks such as diagnostic methods and pulmonary diseases, thus showing the slight shortcomings and methods of improving diagnostic systems, a fact that justifies the possible corroboration of the medical field with that of artificial intelligence. The quintessence of this topic is a medical synthesis that reveals the degree of involvement that must be granted. The second chapter has a more descriptive role regarding the foundation of the developed model that is materialized by the medical data. It focuses first on how the data were prepared for building the model. Once we have the necessary data, the next stage described is that given using the generic architecture approach for binary and multi-class classification. It also introduces the work methodology that denotes different changes to the construction of the model or certain informative decisions such as the number of epochs used. The last chapter refers exclusively to the results obtained, attributing to the related methodology, as well as the obtained conclusions, corroborating our personal opinions regarding them, are presented.

## 2. Materials and Methods

### 2.1. Data Collection and Pre-Processing

The dataset could be equated as an essential component in the use of artificial intelligence techniques for various infectious disease detection and diagnosis. It consists of data collection that includes relevant information about patients, presenting a clinical or epidemiological character, as in the current research, aiming, particularly, at identifying patterns associated with various infectious conditions using their size and diversity.

Throughout the current research, four datasets were integrated into the development process of the models. The desire was to validate the results obtained in a larger area. It is important to mention that not all samples from each were used, considering the limited computational sources and their vast size. As a general approach, during the development of the models, different quantities of samples were used, with these being added gradually for the training. In other words, the size of the databases was varied and they were used to analyze the results of the obtained models step-by-step in the specified context. Broadly, a very relevant aspect is the quality of the database. The number of classes for each is presented in Table 1. The essential characteristic of these is authenticity, to which validity could be also added, which is the most fundamental thing when it comes to such models. Retrieved from other scientific works, the databases are presented as follows:

- CT-COV19 [22]—via IRB approval, CT-COV19 is a dataset of approximately 13,000 non-contrast lung CT scans in which chest cavity volume reconstructions are set at a slice thickness between 0.3 and 1 mm;
- COVID-19-CT [24]—all CT scans are classified into novel coronavirus pneumonia (NCP) due to the SARS-CoV-2 virus, common pneumonia, and normal controls being available globally to help clinicians and researchers fight the pandemic, where COVID-19 is making its presence felt;
- COVID-CT [25]—contains 349 CT samples belonging to 216 patients diagnosed positive for the COVID-19 virus and 397 CT images, with a negative diagnosis for COVID-19 having origins in bioRxiv and medRxiv servers;
- SARS-CoV-2 Ct-Scan-Dataset [26]—contains 1252 positive CT scans for SARS-CoV-2 (COVID-19) infection and 1230 CT scans for patients not infected with SARS-CoV-2, totaling 2482 samples collected from real patients in hospitals in Sao Paulo, Brazil.

It should be noted that within the datasets presented above, the data are stored in the form of PNG format. In other words, they had already gone through certain stages of processing until the moment they were proposed for research. More precisely, their origin is from a 3D volumetric module, where there is a variety of slices. The most significant ones were chosen and proposed, hence their presentation mode, namely 2D. Thus, the algorithm will focus only on a 2D approach. A more advanced approach in the sense of designing a system that starts from the patient’s original data, checking the most relevant slices for the patient’s diagnosis, would be interesting [27]. Thus, the input of the algorithm would be volumetric data, at which point there is a selection criterion of the most significant slices to achieve a correct diagnosis. This is where 3D neural networks for training come into play. Broadly, using 3D CT scans for COVID-19 detection can be beneficial in certain cases, but it also comes with its own set of challenges and considerations. Three-dimensional CT scans provide a wider spatial information about the entire volume of the organ being scanned. This can be especially important in medical imaging tasks where understanding the three-dimensional structure of the affected area can contribute to better diagnosis. At the same time, the sensitivity could be improved. Three-dimensional CNNs can potentially capture more subtle patterns and features that might be missed in a 2D CNN, leading to improved sensitivity in disease detection. On the other hand, there are several challenges in this approach. Processing 3D volumes is computationally more demanding than working with individual 2D slices. Training and inference times may increase, requiring more powerful hardware. Obtaining labeled 3D datasets for training and validation might be more challenging than 2D datasets. Annotating 3D volumes is also more time consuming. Proper preprocessing of 3D CT scans is essential. This includes resampling, normalization, and handling class imbalance in 3D space. In summary, using 3D CT scans for COVID-19 detection can offer benefits, but it requires careful consideration of the challenges and the specific nature of the dataset. Experimenting with both 2D and 3D approaches, and understanding the tradeoffs involved, would be a reasonable strategy.

The rudimentary stage involved in the development of a CNN model associated with solving a classification problem in the field of medical imaging, especially database analysis, is a fundamental step for obtaining a high-performance neural network. After rigorous indispensable scrutiny of the original data, one can proceed to the next step, namely the preparation of a dataset for training and testing the defined model. Correspondingly, it has opted for image processing which deals with the investigation and manipulation of images to extract useful information and improve visual quality or interpretation for the training process of the model.

In the first instance, the resolution of the image was the starting point in this context. Data made available at a higher resolution may require more computing power and memory to process, which may affect the time and performance of the training process. In this work, it was decided to reset it to 224 × 224 px, a value used often in the literature for this kind of problem.

In the context of ML, splitting the data into training, validation, and test sets is a common practice to evaluate a model’s performance while also preventing overfitting. The most frequent training–testing ratio is the percentage split (80-20)% [28]. However, in many cases, a separate validation set is also used to fine tune the model’s hyperparameters. In this paper, the training-testing validation ratio is based on the following distribution: (60-20-20)%. In machine learning, particularly in the context of supervised learning where the data are labeled, the stratified split strategy is used to ensure that the distribution of the target classes remains consistent across different subsets of the data, thus eliminating the possible appearance of data leakage. In this way, the mechanism of dividing the data into the categories specified is feasible via the split-folders function in which the data are distributed automatically into train, validation, and test samples to each corresponding folder. The test set has an important role in checking the performance of the model, thus testing it on data that it has not encountered before.

**Figure 2:**
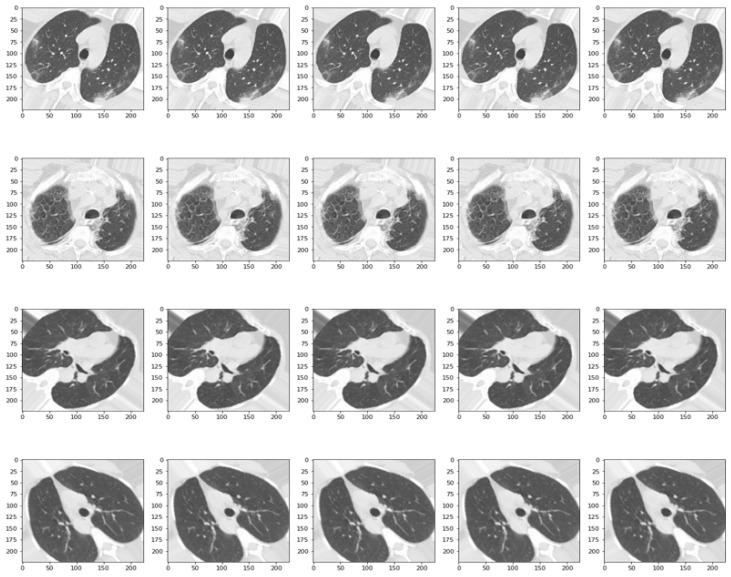
A batch of 20 samples from the resized and augmented SARS-CoV-2 Ct Scan Dataset.

Given the limited number of tomographies, a number given using the medical approach of not constantly exposing patients to such radiation, a technique can be used to increase the size and variability of the samples, namely data augmentation. The purpose of augmentation is to make the model more robust and better generalized by exposing it to more variations and scenarios that it may encounter in real situations. By way of explanation, the object of interest in the image, in this case, the lung cavity, can be placed in different positions so that the algorithm can understand, to some extent, the meaning of the image, as can be seen in Figure 2. In this way, the pixels in the image undergo different changes via specific operations, such as rotation at different angles, translation on the horizontal and vertical axes, shifting, shear range, zoom, and horizontal flip. This approach used an instance of the “ImageDataGenerator” class from the Keras library, which can be used to perform data augmentation on images while training a neural network model. Thus, the scaling factor was assigned a value of 1/255, so that each pixel is divided by 255 to obtain values in the real range [0, 1], a process also called normalization. For the rest of the augmentation parameters, depending on the dataset, several values were tested so as not to degrade the integrity of the data.

A useful approach to enable the convolutional neural model to better identify the specific features of a class is to extract the region of interest from the hypothesis; in the present case, it is the CT scans of the lung cavity. At the current literature stage, it is observed that this approach is less used, preferring to work directly on the entire CT. By this, the pulmonary cavity is extracted from the entire CT with the area outside it being removed. Through this approach, the algorithm can focus strictly on the lung surface, thus increasing the efficiency of the learning process. In a recent study [29], this approach is detailed in an interesting way, applicable via a segmentation process based on a U-Net type network. In comparison with this, within the current work, a segmentation function implemented in Python, without the need to use a neural model, was developed, which gives speed in image processing. The background in such a sample represents the level of absorption of X-rays that do not originate from the anatomical structures in the patient’s body that are being examined. The background appears on the CT image as shades of gray and can vary depending on multiple factors such as machine settings, noise level, accuracy of detection, and image processing technology. Thus, this mechanism to segment the lung area from CT scans to detect areas affected by certain infectious diseases such as COVID-19, using deep learning, presents a variety of advantages. Firstly, the model could allow us to focus on relevant and fine-grained features. This can improve the accuracy of the model and reduce the impact of irrelevant features that can have the effect of hampering the learning process and, by default, incite confusion in future predictions. From another perspective, CT scans usually contain a large amount of information, which can make processing the entire image computationally expensive. By strictly extracting the surface of the lung, the amount of data to be processed is significantly reduced, which can improve the speed and efficiency of the model.

**Figure 3:**
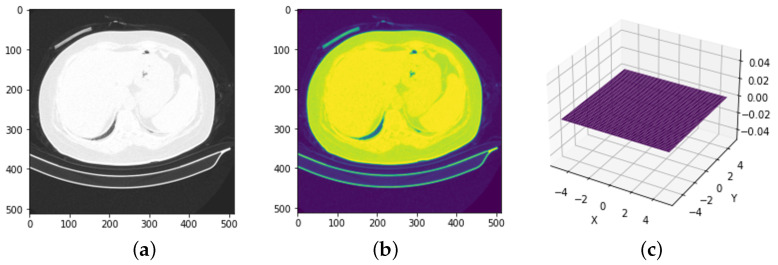
Applying the Gaussian filter to a sample from the COVID-19-CT dataset. (**a**) Original CT. (**b**) Dataset after applying the filter. (**c**) Probability distribution with zero standard deviation.

The implementation of this technique was a gradual one. As a first step in the preparation for extracting the desired area, a Gaussian filter (frequently used as a preprocessing step in image segmentation, edge detection, and feature extraction [30]) is applied to reduce noise in the grayscale image, with a mask kernel size of 5 × 5 and a σ parameter equal to zero, which expresses the standard deviation as shown in Figure 3.

Gaussian filters are often used extensively in various fields, including medical imaging, artificial vision, and signal processing. In particular, they are frequently used as a pre-processing step in image segmentation, edge detection, and feature extraction.

**Figure 4:**
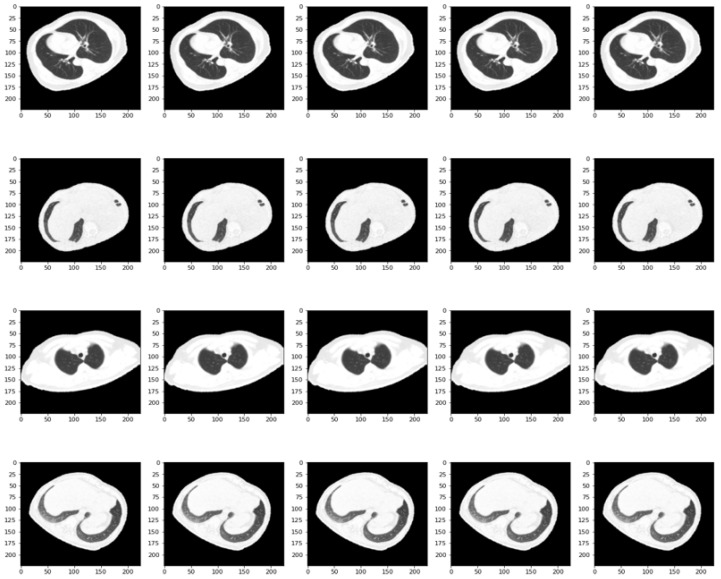
A batch of 20 samples from the resized and augmented COVID-19-CT dataset.

After removing the noise and smoothing the image, the next step is to integrate a threshold value on the images to obtain a binary one. The optimal separation threshold between background pixels and object pixels was obtained by using the Otsu algorithm [31] such that the wavelength is determined automatically. By using this method, the need to set the threshold manually is eliminated. Next, the contours in the binary image are identified. In this way, all the contours are returned in the binary image and the hierarchy between them. Since only the largest contour represents the lung cavities, it is selected by comparing all contours according to their area. To obtain an image showing only the lung cavities, an initially empty mask is created and the largest contour is drawn on this. This will fill the inner area of the outline with white pixels and the rest of the mask will remain black. Finally, a mask is applied to the original image, which will give an image where all pixels outside the lung cavities are black. A result of such an operation at the level of available data is visible in Figure 4. The sequence of steps for preparing this data involves two main operations: extraction and augmentation. The data are extracted and then augmentation takes place.

From a summary perspective, highlighting the area of interest in the image, and at the same time transposing it in different scenarios, can lead to highly accurate results. It is mentioned that all these pre-processing techniques were gradually integrated into the research, taking into account the desire to improve the models. Using this approach, namely centralization and concentration of the algorithm strictly on the area of interest brought improvements to the performance level.

### 2.2. Convolutional Neural Network Conception Methodology

#### 2.2.1. Binary Classification Approach

From a perspective of the categorization of patients, the conception of an architecture in the form of a convolutional neural model, concentrated on solving the problem addressed, gradually starts from the consideration of two classes; the one in which patients suffer from the infectious disease, COVID-19, who will be included in the “COVID” class, and the one in which this condition does not make its presence felt on the patients that will constitute the “Normal” class. Thus, regardless of whether pulmonary characteristics caused by other respiratory insufficiency are noticed in a CT scan, the algorithm shall place the patient in the Normal class. This helps the model to detect unequivocal features of the lung areas affected by COVID-19. To propose a certain architecture with satisfactory performance, several versions of the proposed primitive model were passed. Within them, distinct changes were made at the constructive level of the neural network, videlicet, convolution, MaxPooling, and fully connected layers. Thus, another type of modification was defined by the value changes in the layers, either the number of neurons or the percent of dropouts.

In the first instance, we desire to obtain out own model, without using pre-trained models to gain several important remarks. Appropriately, different structural changes at the architecture level and their impact on performance could be analyzed. In this manner, the first elect dataset, more precisely the SARS-CoV-2 CT-scan dataset, is a balanced one in the number of copies by having 1252 CT scans belonging to the COVID class and 1229 for the Normal class. An equitable dataset is particularly important given the desire to recognize the differences between the classes. In this case, being an early stage, a simplistic architecture was proposed, expecting the number of convolutional layers (32 and 64 neurons convolutional layers) interspersed with those of Max Pooling, dropout (50/75%), and classification ones (512 and 1 neurons dense layers). In general, an appropriate optimization algorithm must be specified for the model compilation stage. In this research, we opted for Adam’s learning rate parameter, which is widely used for its efficiency in various tasks [32]. The by-default value of this learning rate is 0.001 and we decided to not change it during this theme.

**Figure 5:**
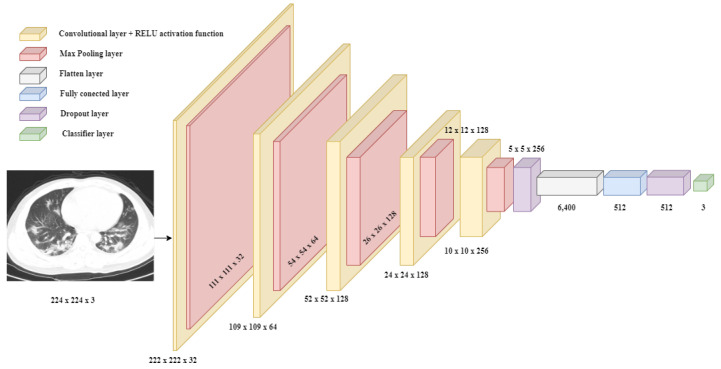
The final neural architecture of the binary CNN model.

In pursuance of generalizing the model and preventing overfitting, a dropout layer, following immediately after the convolutional ones, was adopted, with different percentages, namely 50% and 75%, respectively. The two convolutional layers present 32 and 64 neurons in this order, respectively, of which 512 layers were allocated for the linear classifier. Also, the number of epochs, namely 10, was kept constant during the training performed, and the number of steps was not explicitly set; thus, we let the algorithm choose the best decision concerning the number of samples. After two versions, other techniques were introduced to help in a more in-depth learning of the model, videlicet, and data augmentation via various operations at the pixel level in the images and model checkpoint. These methods were introduced by observing a tendency of the model to reach a possible state of overfitting. This possibility arises from the achievement of performance units that are not exactly feasible. In addition, there is also a mechanism by which the model could not be forced to learn for a rather long period, termed early stopping. During the version change process, a fine-tuning mechanism was chosen for obtaining a better model. Therefore, the saved and loaded model was trained on other datasets, namely CT-COV19 and COVID-CT. This procedure is a common technique used in ML to adapt a model, which has acquired prior knowledge, to a new task or dataset. The basic idea behind this concept, which translates as fine tuning, is to start with a model that has already been trained on a large dataset, then continue training on a smaller dataset that is related to the original but contains some differences, having images as the point of interest that are specific to the new task. The reason fine tuning is effective is that pre-trained models have already learned useful features and representations that are transferable to other tasks or domains. Thus, transferable knowledge can be leveraged to achieve better performance than training a new model from scratch. Particularly, the best-performed achieved architecture was tested on the other datasets to analyze the behavior (Figure 5).

#### 2.2.2. Multi-Class Classification Approach

**Figure 6:**
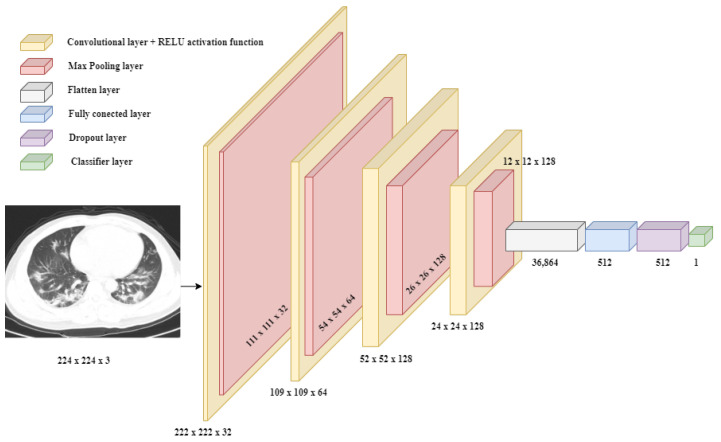
The neural architecture of the three-class classification CNN model.

Since there are many lung diseases in the medical area, as a result, in this project, a new class called “Pneumonia” was added to the existing categories (“COVID”, “Normal”), thus starting from the initial point. This refers to people affected by a normal or viral pneumatic infection and not that caused by COVID-19. By introducing a new class and bearing in mind the existence of some similarities between the negative results caused by pneumonia and COVID-19, a more concrete diagnosis of the patient can be made. In other words, these three distinct classes enable the development of DL models to classify images of a pulmonary factor, whether X-rays or CTs, accurately. The previously presented architecture represents the starting point, towards the construction of other neural models, constituting a structure that presents a higher degree of generalization. To make this transition to the classification type level (from binary to multi-class), certain parametric changes must be taken into account. As a result, at the architecture level, the activation function on the last classification layer will be Softmax, which reveals an important role [33], and the number of output neurons shall necessarily increase to three because it is desired to solve a multi-class classification problem. The best-performed achieved model is presented in Figure 6.

#### 2.2.3. Transfer Learning Approach

Predefined models, within convolutional neural networks, are deep learning models that have already undergone a training process on a large set of data and are available for use in different tasks that can come from several fields, among which medical imaging emerges. In other words, these models are often trained on large datasets and are taught to recognize various features in images such as edges, corners, and various other patterns that can be used for image recognition and classification requirements.

Several approaches pertain to their use. Surrounded by this theme, the primary step in using certain pre-trained models was to download the synaptic weights specific to image and architecture classification problems from the online environment. It should be noted that only the convolutional part has been preserved, and the linear classifier of the initial stage has been updated. In more detail, following the development of models that have achieved satisfactory performance, on a scale that can be proposed as solutions, architectures have been obtained that possess a high-performance linear classifier. This was added to the predefined networks, and by comparison, much more satisfactory results were obtained. More precisely, the most efficient classifier of the initially proposed networks was chosen and integrated into an existing pre-trained model, revealing another way to approach the subject.

According to the literature, there is a wide range of such models, some of which have been specifically used for research purposes on this topic. Throughout the development of the theme, five of these were used to analyze which model achieves the best performance, constituted by various evaluation metrics. All tests were performed on the COVID-19-CT dataset, and the best model was extended to the second available one. Also, the convolution layers were not trained, only the added classifier. Thus, they were tested in the manner specified above the following: DenseNet201, MobileNetV1, ResNet50, VGG16, and VGG19.

**Figure 7:**
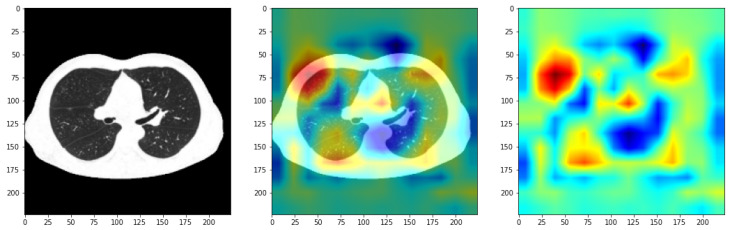
CAM applied to preprocessed COVID-19 CT using VGG16 model.

The goal is actually to generalize the architecture given the limited datasets. During this stage, in which different pre-trained architectures were used, it was also desired to integrate a technique through which the locations in the image, where the algorithm learns the model of the lung cavity affected by the studied diseases, can be visualized more precisely. This is called class activation maps. Technically, it creates a vector of the same size as the activation map produced by the last convolution layer of the model. Further, a traversal of all synaptic weight values associated with the prediction class in the final layer is required. For each activation map, the contribution in the active class is calculated: a contribution is added to the initialized vector that is resized to the size of the image, and a process of normalization of values between 0 and 1 is applied. In the last instance, a pigment distribution is administered to the normalized vector so that the detections can be visualized. Associated with this distribution is the original image, with these being superimposed. An example of the application of this technique is illustrated in Figure 7. The red areas are those that the algorithm considers to belong to a certain class. Thus, we can analyze the coherence of the result provided by the algorithm and the performance achieved in addition to the numerical or graphic evaluation metrics.

## 3. Results and Discussion

In general, the rigorous analysis of the obtained results is of particular importance because it attests to the validity, in this case, of the proposed model. The first method of analysis is the verification of the values of the evaluation metrics of the obtained model. In the current context, this analysis is performed on the test dataset. It derives from the initial dataset as a result of the division into training, validation, and testing data. The test data (on which the decisive analysis is performed) are taken from the initial set with a percentage value of 20%. Given that the algorithm has not encountered this new data before, its effectiveness is highlighted by comparing the achieved performance and relevance with other models as well. This way of distribution is interesting, considering that the algorithm can be put into a new context even though the data are from the same category. Thus, the good results on the test data give us confidence in the model.

### 3.1. Binary CNN Model Outcomes

It should be specified that the approach also has an empirical side. Thus, from the beginning, the methodology was not established in a well-defined way, but several tests were attempted in order to reflect certain useful conclusions. The inception of the model design denotes a condensed character because of the non-involvement of some augmentation operations applied to the original samples so that the pixels do not undergo certain context changes in the first phase, except for the standard resizing to a resolution of 224 × 224 px.

From a structural point of view, the dropout layer was added, between the convolutional and the classifying part of all models in this background. This layer is particularly relevant in generalizing the model, being an indispensable component of architecture. In this case, two percentages usually used were tested on the first two models obtained to analyze their impact. As a result, a higher percentage of it contributes to a decrease in performance, as can be seen from Table 2.

The outset point is represented by version 1.0, which is built on the SARS-CoV-2 CT-Scan-Datase and whose outcomes are presented in Table 2. Considering the multitude of changes at the level of the model and the transition from one dataset to another, with the desire to appreciate their impact, Table 2 contains only relevant ones from which the essential conclusions can be drawn. The metrics’ values of version 1.0 are reached from 498 samples (251 samples for the COVID class and 247 for Normal). It is followed by an attempted modification via adding a new 32-neuron convolution layer (version 1.1). The consequences of the adjustment were verified in terms of metrics and their impact. This addition led to better results and the number of trainable parameters decreased from 95,571,905 to 22,180,833. Therefore, having fewer training parameters, the performance of the model is higher (from an accuracy of 75.30% to an 86.14% accuracy). Furthermore, this new architectural version, namely 1.1, was trained on the other two available datasets, where the best values for the metrics were obtained on CT-COV19, as can be seen in Table 2, on the last two rows. Further, the CT-COV19 dataset was transformed into a binary classification setting, entailing the compression of the initial three classes which encompassed healthy patients and those diagnosed with pneumonia. Thus, these two classes are comprehended as one, namely normal patients. In the current stage, the results are really impressive, but the 99.54% accuracy points to the possible idea of overfitting. So, the values are notable because they are surprisingly high. Within the COVID-CT dataset, the results do not reach the ones related to the SARS-CoV-2 CT-scan dataset and CT-COV19 (72.12% accuracy, 76.41% F1 score), but, in this context, the number of samples is really low. So, the model could have not had time to learn, having a rather limited number available. On the other hand, a generic idea is concretized and consolidated. In all cases, the effect of the dropout layer with a higher percentage is to decrease the performance but increase the generalization of the model as it resulted, also, from the later version 1.2. Here, two special techniques were integrated via which the model could be saved in an h5 file (model checkpoint) and the overfitting could be avoided (early stopping). Now, the data were augmented using specific pixel operations and all metrics have decreased by about 0.2%. This decrease could be determined, at the same time, by either adding a new 128 convolutional layer, or adding a new 25% dropout and changing the old one into 25%. Within the third variant, by adding more neurons on the fourth layer, from 128 to 256 neurons, the performance increased. As a result, the number of trainable parameters has increased surprisingly by 10 times. Also, all the performances improved. In a last attempt to determine the best model, in addition to the types of structural changes, the fine-tuning technique was used, passing the saved model through the other two sets of data available, leading to the performances surprisingly increasing. Otherwise, it is no longer necessary to build the model sequentially but only to load it. It was noticed that the CNN network should contain layers with more neurons on the last convolutional ones and the inception to be with fewer neurons. As a consequence of all these modifications approached, it achieves the following metrics: 95.15% accuracy, 95.86% precision, 92.20% recall, 98.82% AUC, and 93.99% F1 score. Taking into account a more meticulous approach that is accumulated from passing through several datasets, architectural changes, and the integration of techniques that denote a higher degree of robustness for the model, these evaluation metrics seem satisfactory. These tend to reach current evaluation metrics in the literature [34].

**Figure 8:**
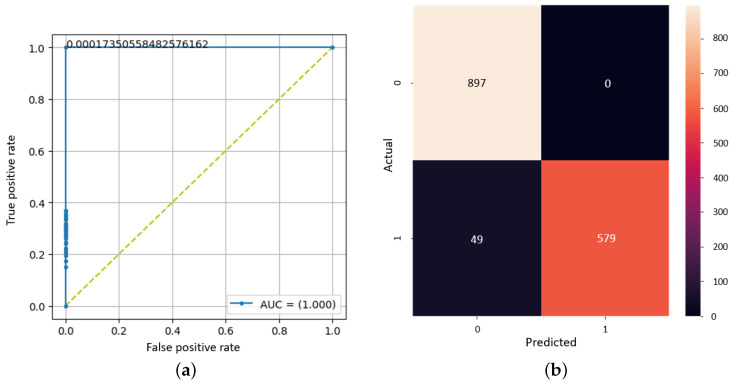
The results of the binary model proposed on the COVID-19-CT dataset. (**a**) ROC plot. (**b**) Confusion matrix.

Further, more detailed research on the level of performance is desired. In this way, the study of the confusion matrix can be chosen. Bearing in mind that the prediction is, in fact, a subunit number, it is necessary to establish a threshold value for separating the classes. A method of choosing the optimal threshold value comes from an ROC chart. This is a graphical representation of the performance of a binary classification model. Moreover, it could be defined as a plot of the true positive rate versus the false positive rate for different classification thresholds. To create an ROC plot, the output probabilities of the model are sorted from highest to lowest and a threshold is set to decide which class an instance belongs to. By varying the threshold, the true positive rate and false positive rate can be calculated for each threshold. In general, a standard value can be used, namely 0.5, for the threshold, but it has been observed that it is much more efficient to use a similar method of automatically establishing this value, given the results at the level of the confusion matrix. In Figure 8, the value obtained from the ROC (around 0.0001735) is the best threshold to predict the classes, resulting in the confusion matrix having the highest values for true positive and false positive cases.

### 3.2. Multi-Class CNN Model Outcomes

The immediate transition of an architectural nature only implies certain parametric changes and, at the same time, the revision regarding the use of databases, bearing in mind the fact that a three-class classification problem must be solved which arises from the gravity of the problem. Therefore, at the level of the CT-COV19 dataset, the original division of classes was returned, separating, in this way, pneumonia and healthy patient samples. In general, within this approach, it was decided to keep the same methodology as in the previous version. Certainly, there are some differences. For compiling the model, the loss is now categorical, not binary cross-entropy anymore. After adopting all the necessary changes, synthetically, at an approach level, the first model obtained here on the CT-COV19 set, which achieved a performance of a 92.26% accuracy on testing from the 1252 samples, was transferred further to the COVID-19-CT, where the entire architecture with synaptic weights was discarded. Within this dataset, there are 2700 copies equally distributed for each class, therefore possessing 900. The metrics’ values are good for such a quantity of data, reaching a 96.78% accuracy, 96.74% recall, and 96.83% F1 score. Once the initial architecture was tested on the new dataset, the idea of the architectural modification directly on the h5 file was followed. In other words, it opted to add a dropout layer between the linear classifier and the convolutional sequence straight into the discarded file. This technical idea is very useful for increasing the training speed and consolidating the knowledge of the model. The generalization of the model can be improved via a repeated training process, as our brain is used to. Once the model is saved in such a file, it is possible to opt for adding new layers and resuming the training process so that new knowledge can be acquired and old ones can be deepened, and by adding techniques such as early stopping, overfitting does not become an integral factor for the model. So, the percentage of the dropout layer is 50%, and a decrease in performance was observed by approximately 0.02%, which is expected considering the increase in generalization. Last but not least, based on the accumulated experiences, a new architecture was built, wanting to obtain higher performances because the dataset is wider. The latter proved to be the most efficient and the best from the point of view of all the evaluation methods of the model, namely 1.4.2.1, which can be seen in Table 3.

Thus, after a multitude of experiments and various attempts, this architecture was obtained, namely version 1.4.2.1. The way of conceiving such convolutional structures is interesting, opting for the sequential approach. This method is very beneficial due to the control over the entire model. The method of adding layer by layer can lead to a beneficial gain of experience by analyzing the results step by step.

**Figure 9:**
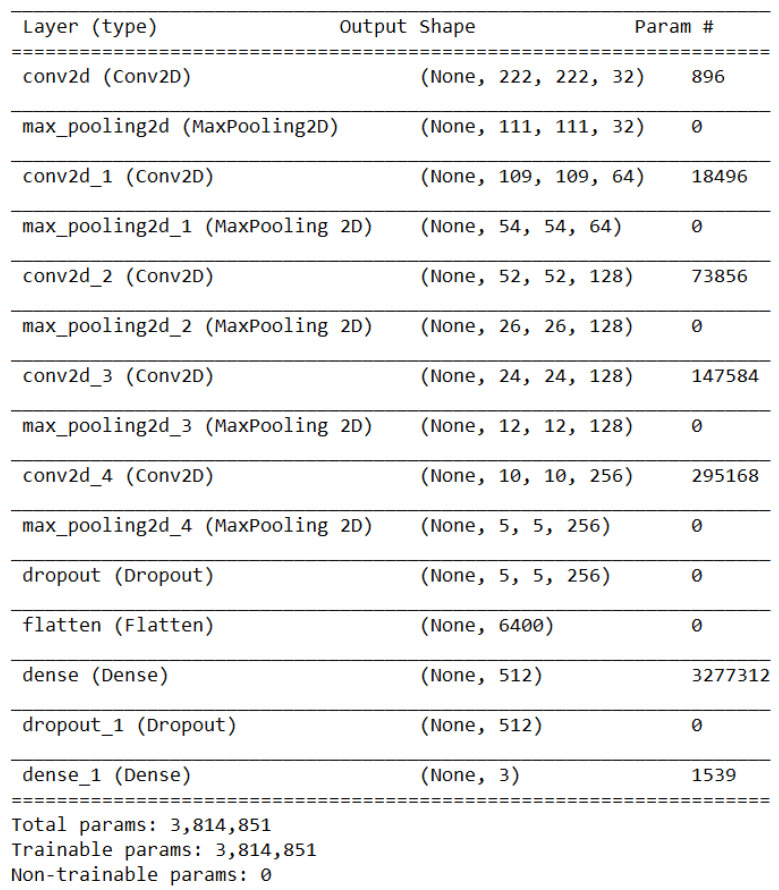
Best performing sequential multi-class model.

In Figure 9, all the layers of the previously specified model are presented. The first part is represented by the sequence of 2D convolutional layers with those of MaxPooling. It can be noted that the most training parameters for this section of the network are on the last layer, due to the fact that it also has the most neurons. Then follows the dropout layer so that the algorithm forgets part of what it learned to achieve classification within the dense type layers, where there is another such layer for dropping knowledge. This structure for an image classification problem can be extended to several other partial study cases, due to the flexibility of CNN models.

**Figure 10:**
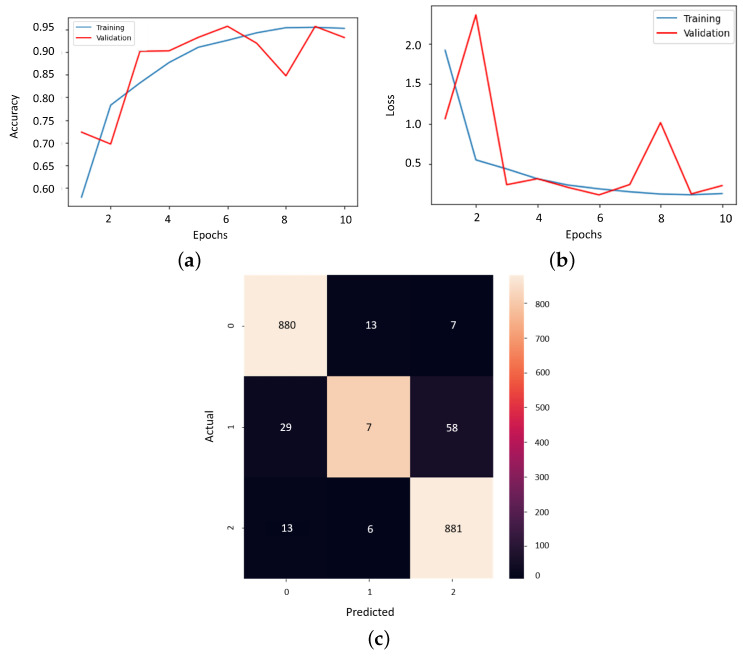
The results of the multi-class model conceived on the COVID-19-CT dataset. (**a**) Training curve. (**b**) Loss curve. (**c**) Confusion matrix.

The final model from this stage must be meticulously analyzed considering the results provided, which is visible in Figure 10. The training curve and the loss curve give the linear change along the 10 epochs in the training and validation stages. What is essential here is the convergence of the algorithm which seems to be almost reached. Also, the confusion matrix thus involves a new label associated with a patient suffering from pneumonia. On the main diagonal, all the correctly detected cases can be found.

### 3.3. Transfer Learning Model Outcomes

For this stage, we chose to change the convolutional part using a predefined network. So, the transferable character refers to the fact that a combination is made between what already exists in the literature and what has been developed so far.

The initial stage starts with a sample of 15,000 copies that the COVID-19-CT database possesses by using DenseNet201. In this case, via the architectural changes, it was observed that reducing the number of neurons leads to better performance, with the model achieving an accuracy of 94.67%, a precision of 94.69%, and an F1 score of 94.65% from 3000 samples, which represent the test data. Next, more samples were introduced, reaching 17,397. So, the 20% test data become 3483. In this way, several networks were introduced for analysis: ResNet50, VGG16, VGG19, and MobileNet.

**Figure 11:**
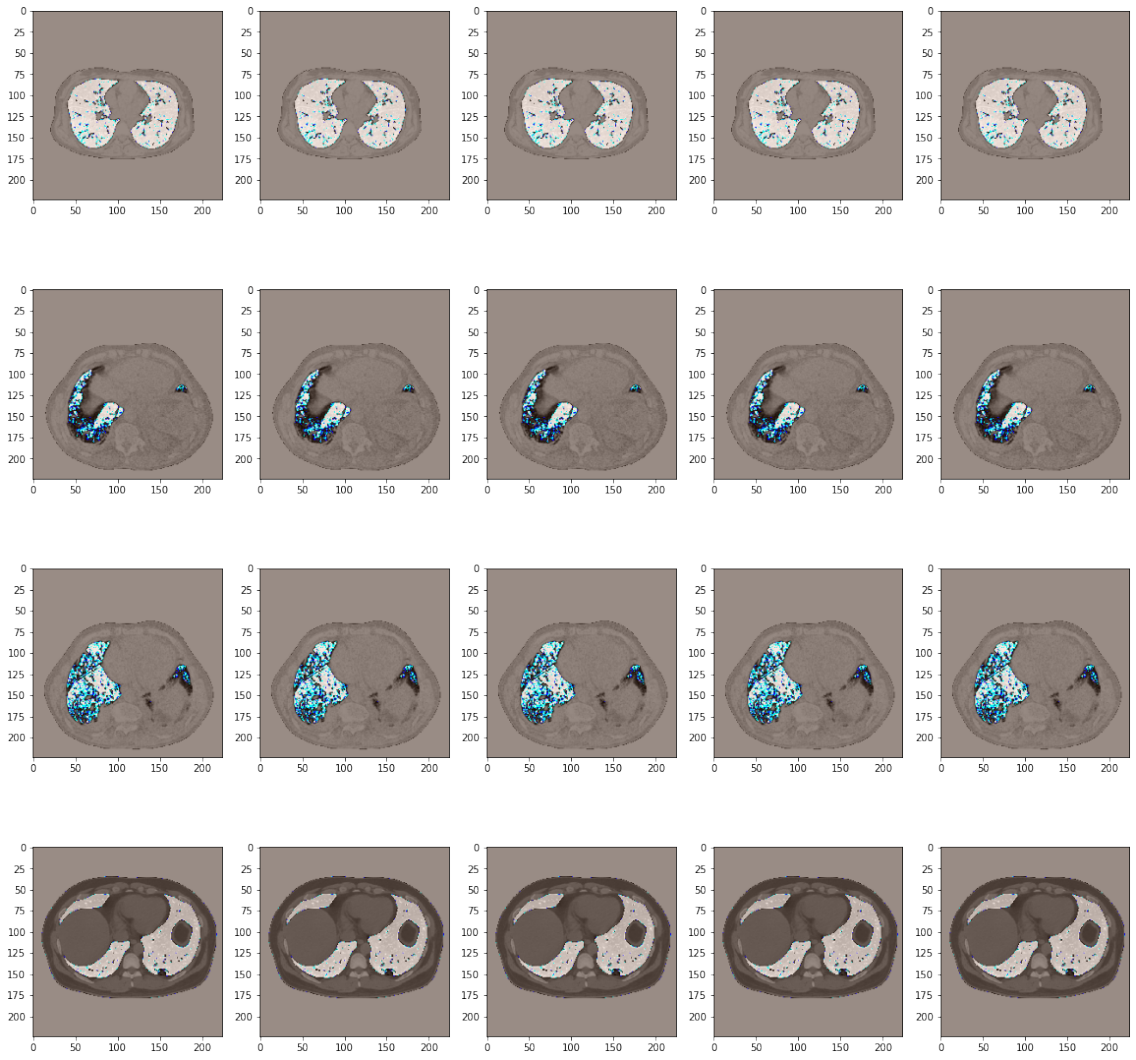
Pre-processed data using VGG19 pre-trained model.

An interesting approach in the case of using such models is the fact that data pre-processing methods can be used. These models contain specific functions that perform different operations at the pixel level. An example of samples processed by such network operations can be found in Figure 11. In the attached figure, once the extraction technique is performed, the processing stage corresponding to the VGG19 network is also applied. This led to an increase in performance, from an accuracy of 96.76% to 97.70% out of 3483 samples.

During this stage in which the transfer learning technique was used, different existing models were tested, wanting to highlight the one that achieves the best performances. The results of the evaluation metrics for each model are presented in Table 4. These metrics are obtained from the test data specified above, so the number of samples reaches up to 3483 for each class.

**Figure 12:**
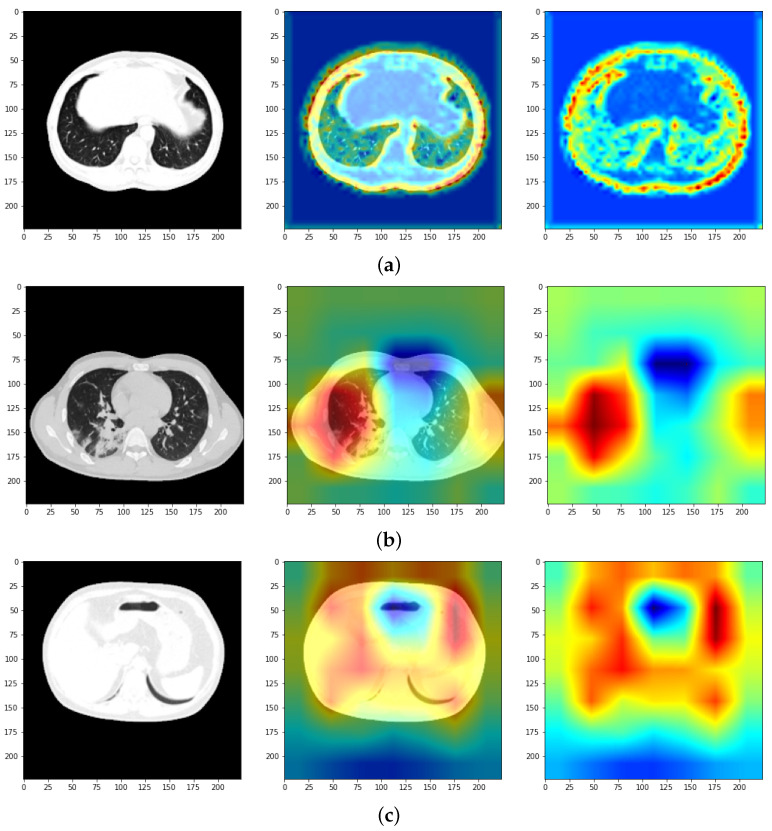
Applying CAM over samples. (**a**) MobileNetv1, (**b**) ResNet50, and (**c**) VGG19.

All the architectures present satisfactory values and thus, a justified differentiation is desired via an analysis at the detection precision level feasible by using the CAM technique. In the literature, one can observe the tendency to skip this step, leaving the evaluation of the model in principle to the numerical metrics. Using this mechanism, the algorithm’s learning mode can be accurately visualized. Struggling with a problem based on medical imaging, it must be visualized if the algorithm tends to learn the exact locations of the lung anomalies caused. Thus, we desire to identify a network that possesses precise detection. Surprisingly, such a network is not so used in the context of the current theme. Among all the models designed, the one based on the MobileNet network stood out, due to the precise detection of the features in the image, as can be seen in Figure 12, compared, for example to ResNet50 and VGG19. It can be observed that this model is the only one that focuses strictly on the pulmonary cavity, which is also the object of the study. Instead, the other networks tend to focus on the entire area of the cavity, but not in as much detail as the MobileNet model.

Having chosen the MobileNet network, the last step in obtaining an efficient and applicable model is ensuring convergence. Throughout the training, a tendency of the model to a longer training to reach convergence was seen. So, at the level of the training process, 20 epochs were chosen with the aim of reaching the convergence of the algorithm compared to the models presented previously, where the possibility of continued learning by the algorithm is observed. From a structural point of view, the classifier contains two layers of 512 neurons each, and also the last output possesses three. This choice was made on the basis of some experiments in which we attempted to add more dense-type starts with 1024, respectively 512 neurons, starting from the initial proposed model related to the previous chapter (the multi-class model). It was observed that the results are better based on fewer neurons divided into two layers. The dataset is made up of COVID-19-CT, with 3483 samples available for analysis. The performances are quite impressive considering the number of test samples, namely 0.0751 loss, 97.44% accuracy, 97.58% precision, 97.42% recall, 99.70% AUC, 97.50% F1 score. By comparison with the most recent studies [35], the proposed model is a little lower in terms of the values of the evaluation metrics, but it can be distinguished by the performances achieved at the level of a multitude of other evaluation methods and by the impressive detection precision rendered using the CAM technique.

**Figure 13:**
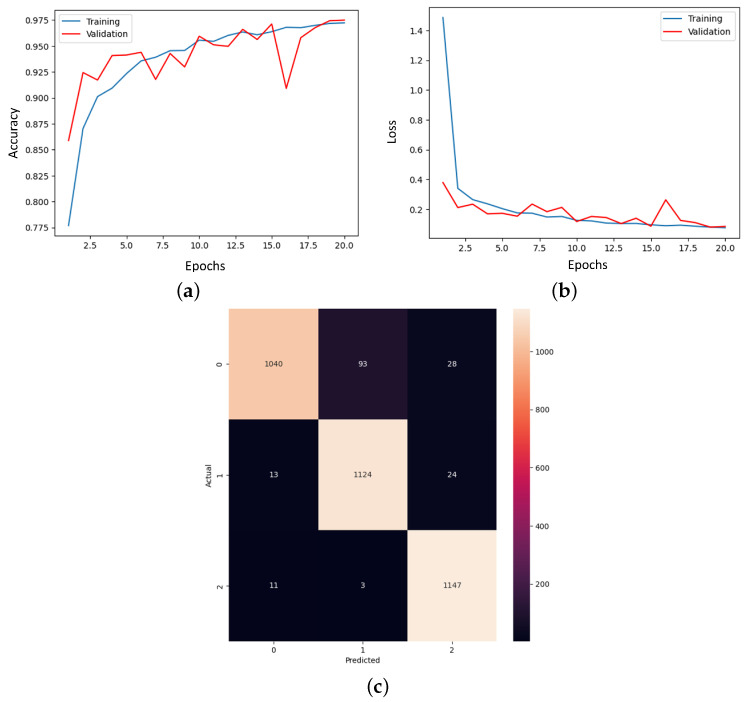
The results of the final proposed CNN model for detecting and diagnosing patients. (**a**) Training curve, (**b**) loss curve, and (**c**) confusion matrix.

Also, the analysis of the final result cannot be missing to demonstrate the effectiveness of the model. The training curve and the loss curve ensure the convergence of the algorithm, and the confusion matrix shows us the high number of correctly detected samples, as could be seen in Figure 13.

## 4. Conclusions

The main contribution of this paper is to develop a new CNN model as a diagnostic tool for medical staff in the context of a clinical problem associated with the consequences of lung diseases, among which COVID-19 and viral pneumonia could be mentioned. On the other hand, it is also desired to present a different approach by integrating a combination of several current techniques in the literature, to illustrate a more complete approach to obtain relevant and reliable results. Encompassed by the many existing methods for such an issue, artificial intelligence applications based on deep learning stand out. Thus, integrating DL techniques in medical imaging can solve many shortcomings. Methodologically, starting from a simple architecture via the layers, a model based on the pre-trained MobileNet network was developed, which achieves a superior performance given by an accuracy of 97.44%, which is a satisfactory value along with other evaluation metrics. High sample numbers significantly improve the algorithms’ ability to learn patterns, especially in identifying lung anomalies during model development. This is supported by data augmentation, which provides the algorithm with different contexts for the objects of interest in the image, which, for this theme, is the pulmonary cavity. The model’s effectiveness relies on maintaining the integrity of the image, ensuring that useful information is not lost. The design involves rigorous analyses to ensure its application. A lung cavity extraction technique allows for focused learning on the area of interest, leading to increased evaluation metrics and faster learning. The model’s results can also be analyzed using the CAM technique, enhancing its efficiency. This approach is crucial for achieving better results in medical imaging. More precisely, the areas based on which the algorithm performs classification can be found outside the area of interest. From an architectural perspective at the level of the neural network, it is better to insert more neurons on a certain layer, thus achieving their condensation which allows for the number of training parameters to increase and the values of the metrics as well. A special role is played by the dropout layer, which decreases performance but increases generalization. Another relevant finding is based on the effectiveness of pre-trained networks. Among all the networks used for the study (ResNet50, DenseNet201, VGG16, VGG19, and MobileNetV1), the most performing from the point of view of all evaluation methods, including CAM, is MobileNetV1, which has an impressive CT detection accuracy despite the fact that it is not often used. The identification process demonstrated a valuable skill in detection precision, posing potential for further research in the current theme.

## Figures and Tables

**Table 1:** The number of classes for datasets.

Dataset	Nr.	Category Class			
**Name**	**Classes**	**COVID**	**Non-COVID**	**Pneumonia**	**Normal**
CT-COV19	3	✓	✗	✓	✓
COVID-19-CT	3	✓	✗	✓	✓
COVID-CT	2	✓	✓	✗	✗
SARS-CoV-2 Ct-Scan	2	✓	✓	✗	✗

**Table 2:** The metric values using unaugmented data.

Version and	Metrics’ Values					
**Dropout**	**Loss**	**acc.**	**prec.**	**Recall**	**AUC**	**F1 Score**
1.0 (50%)	1.1437	0.7530	0.7183	0.8259	0.8257	0.7683
1.0 (75%)	1.7888	0.6165	0.6111	0.6235	0.6953	0.6172
1.1 (50%)	0.0234	0.9954	0.9952	0.9936	0.9975	0.9943
1.1 (75%)	0.0611	0.9882	0.9751	0.9968	0.9949	0.9858

**Table 3:** The metric values of multi-class models.

Model	Metrics’ Values					
**Version**	**Loss**	**acc.**	**prec.**	**Recall**	**AUC**	**F1 Score**
1.4.1.0	0.02139	0.9226	0.9267	0.9207	0.9861	0.9263
1.4.1.1	0.0923	0.9678	0.9692	0.9674	0.9963	0.9683
1.4.2.0	0.3257	0.9300	0.9324	0.9293	0.9792	0.9308
1.4.2.1	0.1324	0.9533	0.9596	0.9511	0.9939	0.9554

**Table 4:** The metric values for pre-defined models.

Model	Metrics’ Values					
**Version**	**Loss**	**acc.**	**prec.**	**Recall**	**AUC**	**F1 Score**
DenseNet201	0.2193	0.9467	0.9469	0.9460	0.9870	0.9465
MobileNetV1	0.1007	0.9615	0.9635	0.9612	0.9970	0.9623
ResNet50	0.0826	0.9701	0.9707	0.9699	0.9975	0.9750
VGG16	0.0831	0.9676	0.9686	0.9653	0.9978	0.9669
VGG19	0.0855	0.9758	0.9713	0.9710	0.9970	0.9707

## Data Availability

The data used as input in this article is based on four public databases so there is no need for special access request. These can be found on the following links: **COVID-CT** (https://github.com/UCSD-AI4H/COVID-CT), **SARS-COV-2 Ct-Scan Dataset** (https://www.kaggle.com/datasets/plameneduardo/sarscov2-ctscan-dataset), **Covid-19-CT** (https://www.kaggle.com/datasets/bayazjafarli/covid19-covidpneumanianormal-cases) and **CT-COV19** (https://github.com/m2dgithub/CT-COV19) all accessed on 22 August 2023.
